# Draft genome sequence data of *Gordonia hongkongensis* strain EUFUS-Z928 isolated from the octocoral *Eunicea fusca*

**DOI:** 10.1016/j.dib.2022.108076

**Published:** 2022-03-22

**Authors:** Jeysson Sánchez-Suárez, Luis Díaz, Javier Melo-Bolivar, Luisa Villamil

**Affiliations:** a School of Engineering, Universidad de La Sabana, Chía 250001, Colombia; b Bioprospecting Research Group, School of Engineering, Universidad de La Sabana, Chía 250001, Colombia

**Keywords:** Actinobacteria, Marine actinomycete, Rare actinobacteria, Corynebacteriales

## Abstract

Octocorals are among the most prolific sources of biologically active compounds. A significant part of their specialized metabolites richness is linked to the abundance of their associated microbiota. Consequently, research on the bioprospecting potential of microorganisms associated with these marine invertebrates has gained much interest. Here, we describe the draft genome of *Gordonia hongkongensis* strain EUFUS-Z928 isolated from the octocoral *Eunicea fusca*. The genome was assembled *de novo* from short-read whole-genome sequencing data. Additionally, functional annotation of predicted genes was performed using the RAST tool kit, including genome mining for specialized metabolite biosynthetic gene clusters using the antiSMASH v6.0 tool. The genome sequence data of *G. hongkongensis* EUFUS-Z928 can provide information for further analysis of the potential biotechnological use of this microorganism and guide the characterization of other related actinobacterial isolates. Likewise, this information increases the analytical capacity for studying the genus *Gordonia*.

## Specifications Table

**Table:** 

Subject	*Biological sciences*
Specific subject area	*Biotechnology, Microbiology: Bacteriology, Omics: Genomics*
Type of data	Table Figure Draft genome sequence data
How the data were acquired	Whole-genome sequencing using Illumina NovaSeq 6000 platform for short reads
Data format	Raw Analyzed
Description of data collection	Strain EUFUS-Z928 was isolated from the octocoral *Eunicea fusca*. High-quality DNA was extracted and sequenced using Illumina NovaSeq 6000 (short reads). Raw paired-end reads were *de novo* assembled following the Shovill pipeline. The assembly was scaffolded with the MEDUSA algorithm, and annotation was performed using PATRIC web resources. Detection of specialized metabolite biosynthesis gene clusters was conducted with the antiSMASH tool.
Data source location	• *Institution: Universidad de La Sabana* • *City/Town/Region: Chía, Cundinamarca* • *Country: Colombia* • *GPS coordinates for collected samples: 11°15′02.1″N 74°13′16.0″W*
Data accessibility	Repository name: OSF Data identification number: R4UZ8 Direct URL to data: https://osf.io/r4uz8/.

## Value of the Data

• The draft genome data of *Gordonia hongkongensis* strain EUFUS-Z928 provides valuable information for the study of the evolution of the genus *Gordonia* and its biotechnological potential.
• These data are valuable for environmental and clinical microbiology, bioprospecting, and biotechnology researchers.
• These data can be used for genome mining to discover novel metabolite biosynthesis pathways.
• Given the potential shown by *Gordonia* species in bioremediation, these data serve to conduct comparative genomics work further and allow a better understanding of the mechanisms involved in bioremediation processes.

## 1 Data Description

The strain EUFUS-Z928 was isolated from the octocoral *Eunicea fusca* collected in Santa Marta Bay, Colombia. Table 1 shows the results of the *de novo* and scaffolded genome assembly of the strain EUFUS-Z928. Scaffolding substantially improved the assembly by reducing the number of contigs by 76.23% and leaving an L50 and L75 of 1 (N50=5,295,384). The scaffolding was performed using as reference the genomes of the closest relatives according to the overall genome relatedness indices (OGRI) results obtained on the *de novo* assembly (Table S1: https://osf.io/q8xus/).

**Table 1:** Characteristics of the *de novo* assembly and scaffolded genome of strain EUFUS-Z928.

Features	*de novo* Assembled Genome	Scaffolded Genome
Genome size (bp)	5,329,221	5,333,421
Total number of contigs	122	29
Largest contig (bp)	599,980	5,295,384
N50 (bp)	252,700	5,295,384
N75 (bp)	105,922	5,295,384
L50	7	1
L75	15	1
GC (%)	67.97	67.96

**Fig. 1:**
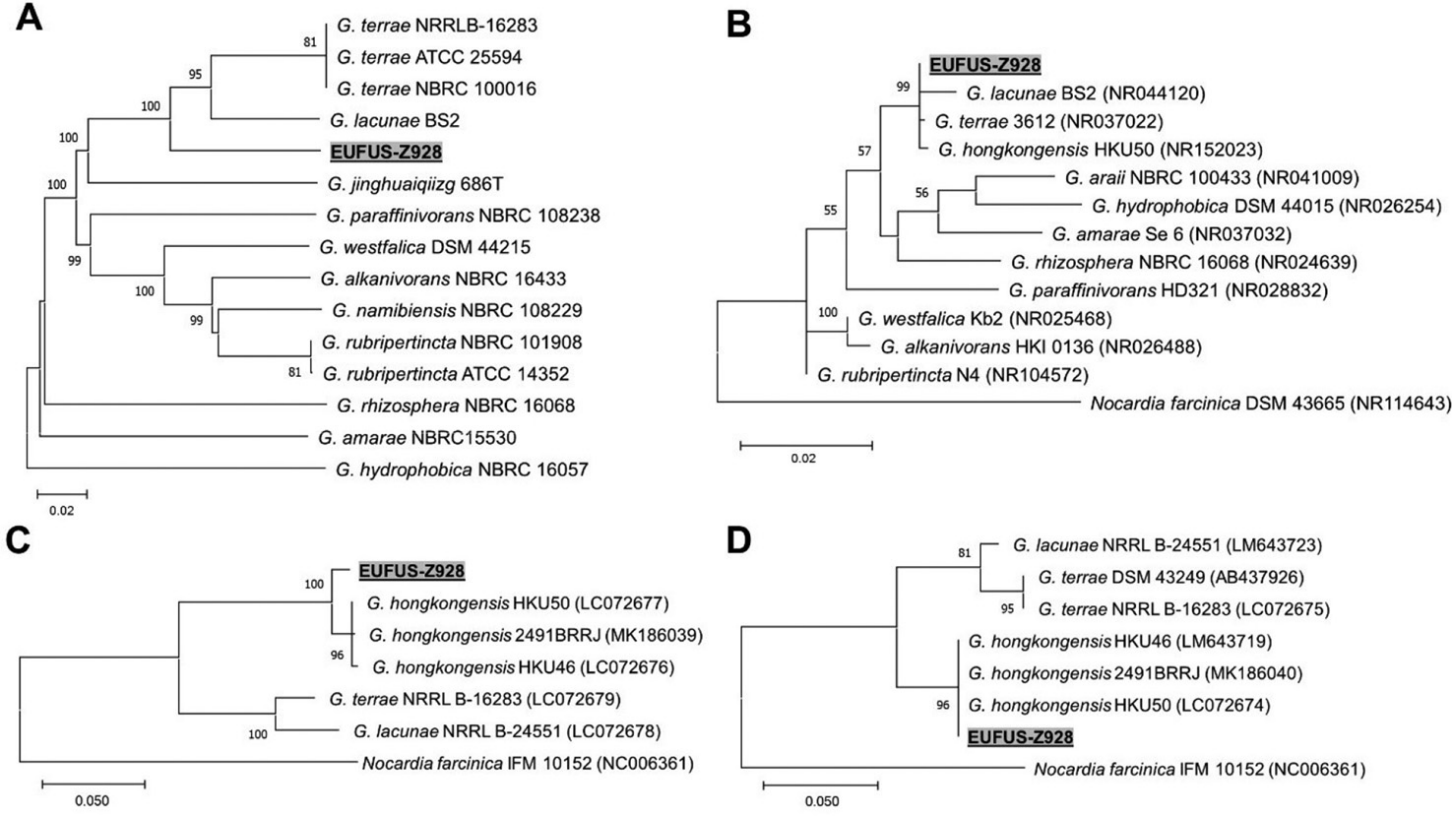
Phylograms of strain EUFUS-Z928 based on (A) whole-genome sequences, (B) 16S rRNA gene sequences, (C) gyrB gene sequences and (D) secA1 gene sequences. The phylogenetic trees were drawn to scale, with branch lengths measured in the number of substitutions per site. The percentage of bootstrap replicates >50% (out of 100 for whole-genome and out of 1000 for single-gene trees) that supported each node are shown. Genome BLAST Distance Phylogeny approach was used for the whole-genome cladogram using the TYGS server. Single-gene phylogenetic trees were inferred by maximum likelihood with the IQ-TREE algorithm.

The genome-based classification and identification found the strain EUFUS-Z928 to be closely related to *Gordonia terrae* and *Gordonia lacunae* type strains (Table 2, Fig. 1A). Phylogeny analysis with the 16S rRNA gene also found a close relationship with *Gordonia hongkongensis* (Fig. 1B). Finally, phylogenetic analysis with the sequences of the genes coding for protein translocase subunit SecA1 (secA1) and DNA gyrase subunit B (gyrB) allowed classification of strain EUFUS-Z928 as *G. hongkongensis* (Fig. 1C and D). It is important to clarify that at the time of the analysis, *G. honkongensis* genomes were not available in the Type Strain Genome Server (TYGS); therefore, it was impossible to include them in the whole genome-based phylogram.

**Table 2:** Overall genome relatedness indices (OGRI) between EUFUS-Z928 and the closely related type strain genomes.

Strain	dDDH^a^ (d0, in %)	dDDH^a^ (d4, in %)	dDDH^a^ (d6, in %)	G+C_Δ_^b^ (in %)	ANIb^c^ (%)	ANIm^c^ (%)
*G. terrae* NRRL B-16283	70.80	34.50	61.50	0.15	87.68	88.83
*G. terrae* NCTC 10669	70.80	34.50	61.40	0.15	87.70	88.81
*G. terrae* NBRC 100016	70.40	34.40	61.10	0.12	87.69	88.83
*G. lacunae* BS2	69.40	35.50	61.00	0.12	88.09	89.19

a digital DNA–DNA hybridization (DDH): formula d0 (length of all high-scoring segment pairs (HSPs) divided by total genome length), formula d4 (sum of all identities found in HSPs divided by overall HSP length), formula d6 (sum of all identities found in HSPs divided by total genome length).

b G+C content difference.

c Average nucleotide identity based on BLAST (ANIb) and MUMmer (ANIm).

**Fig. 2:**
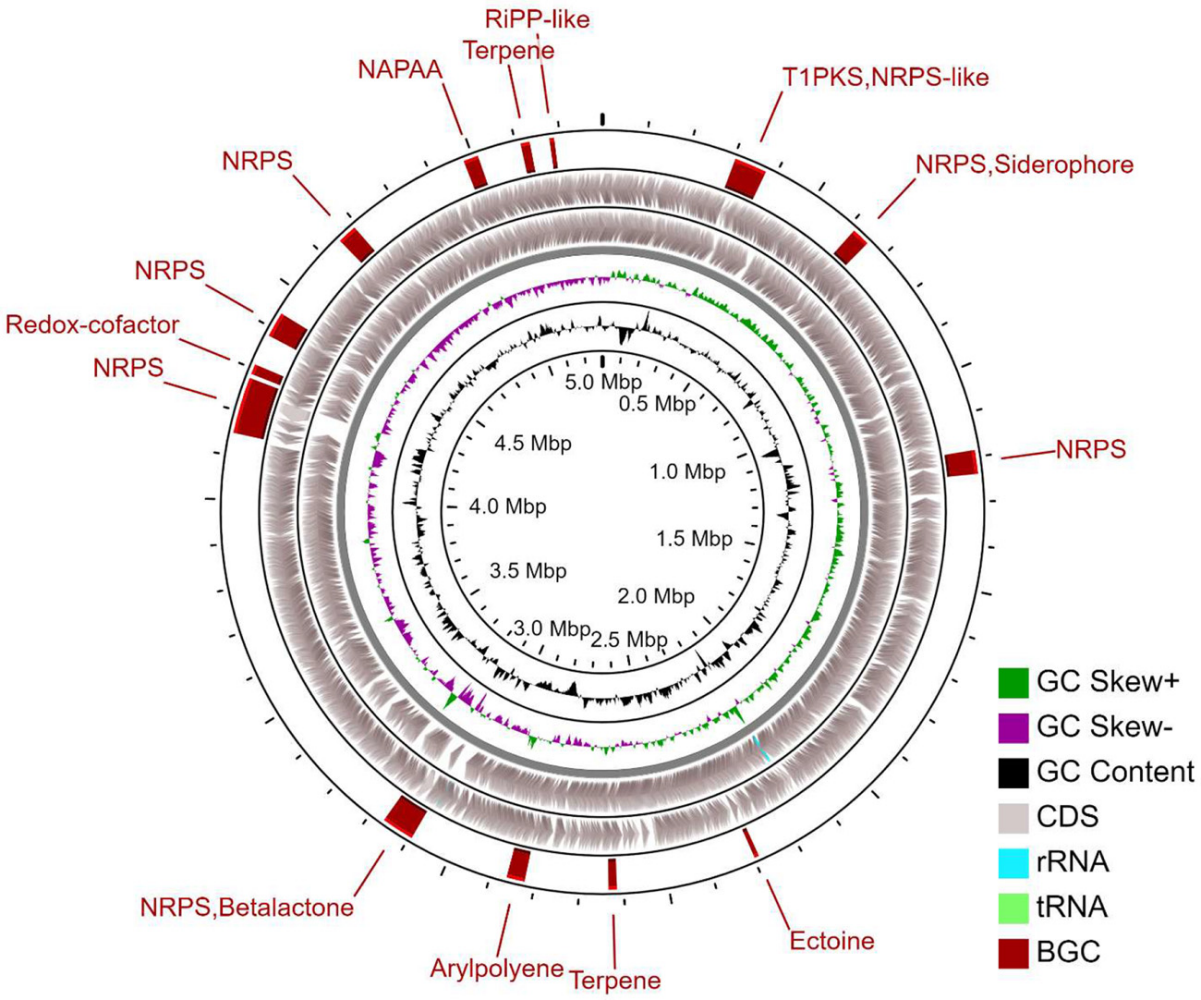
Circular genome view of *G. hongkongensis* EUFUS-Z928. The inner ring shows the length of the genome. The following two rings show the GC content and GC skew, respectively. The gray rings correspond to the CDSs annotated by the RAST tool kit in each DNA direction. The outer ring indicates the BGCs annotated by antiSMASH v6.0.

A total of 5042 genes were annotated in the genome of *G. honkongensis* EUFUS-Z928 (the complete annotation data can be found in Table S2: https://osf.io/2ra3k/). Of these, 4987 corresponded to coding sequences (CDS), most of them (62.44%) with a functional assignment (Table 3). Additionally, analysis with the antiSMASH v6.0 tool identified 14 biosynthetic gene clusters (BGCs) (Table 3 and Fig. 2), among which NRPS and Terpene had more than 1 cluster (i.e., 4 and 2, respectively).

**Table 3:** Annotation results of the *G. hongkongensis* EUFUS-Z928 genome.

Feature	Values
tRNA^a^	47
rRNA^a^	8
CDS^a^	4987
Hypothetical proteins	1873
Proteins with functional assignments	3114
Proteins with GO assignments^a^	991
Proteins with Subsystem assignments^a^	1640
BGC^b^	14
Arylpolyene	1
Ectoine	1
NAPAA	1
NRPS	4
NRPS, Betalactone	1
NRPS, Siderophore	1
Redox-cofactor	1
RiPP-like	1
T1PKS,NRPS-like	1
Terpene	2

a According to the RAST tool kit using the PATRIC service center.

b According to the antiSMASH v6.0 tool.

**Fig. 3:**
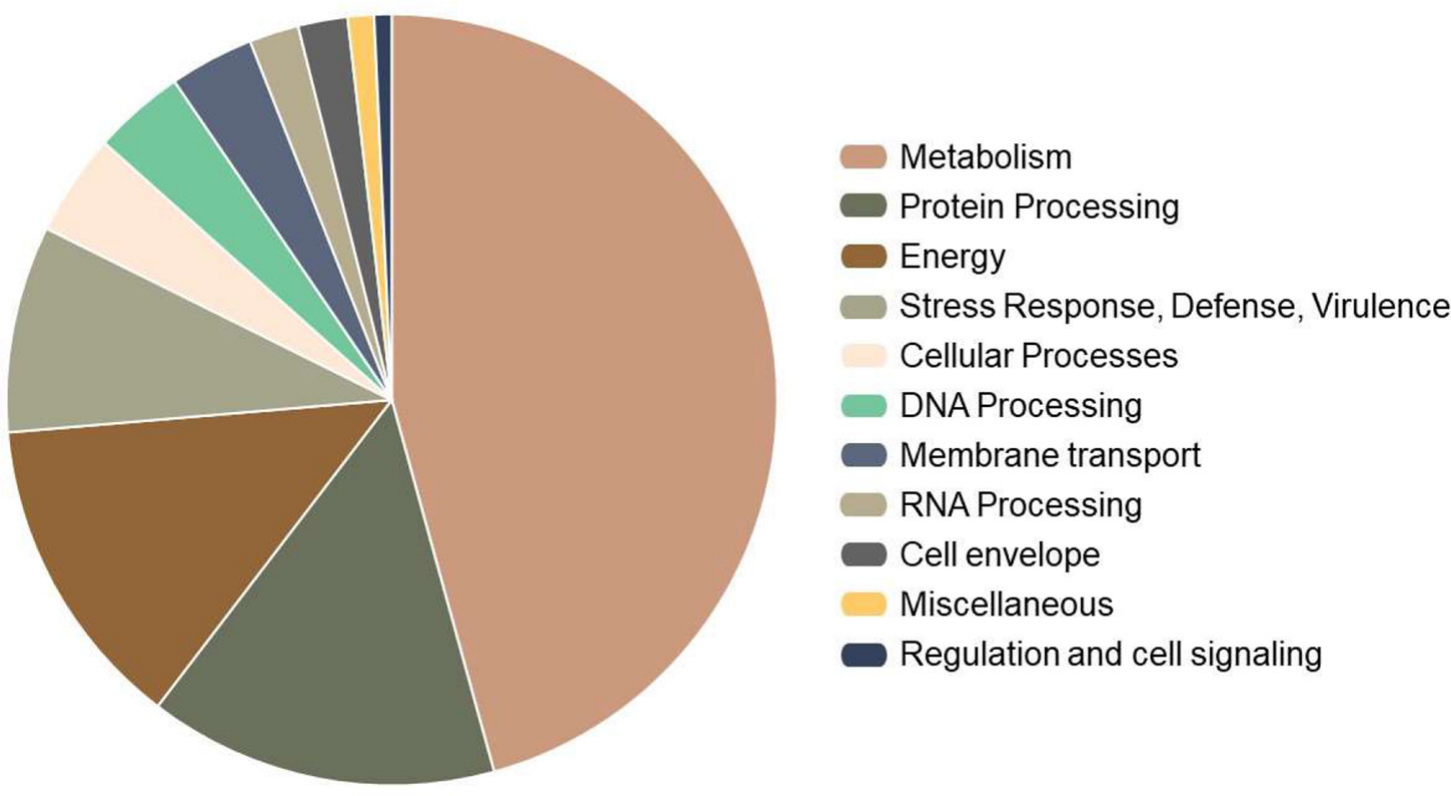
Overview of the assignments to functional subsystems of the *G. hongkongensis* EUFUS-Z928 genome according to the PATRIC annotation service.

Regarding proteins with assignments to subsystems, as shown in Fig. 3, Metabolism (45.61%), Protein Processing (14.76%), Energy (13.23%), and Stress Response, Defense, Virulence (8.72%) were the subsystems with the highest assignments. In the latter, genes related to antibiotic resistance (*n* = 43), arsenic resistance (*n* = 5), as well as genes related to protection against oxidative stress such as mycothiol (*n* = 10) and protection from reactive oxygen species (*n* = 3) stand out. Complete information on the 1640 genes assigned to subsystems is shown in Table S3 (https://osf.io/6j5zs/).

According to the List of Prokaryotic names with Standing in Nomenclature, 47 species of the genus *Gordonia* have been reported so far (https://lpsn.dsmz.de/genus/gordonia; consulted on 04/02/2022). Although several strains of *Gordonia* are opportunistic pathogens, their potential for bioremediation of polluted environments [1] makes them a valuable biological resource in several research areas. The whole-genome sequence and functional annotation data of *G. hongkongensis* EUFUS-Z928 provides valuable information to facilitate the design and execution of more in-depth studies such as comparative genomics and genome mining.

## 2 Experimental Design, Materials and Methods

### 2.1 Strain isolation and DNA extraction

Strain EUFUS-Z928 was isolated from a sample of the octocoral *Eunicea fusca* (collected by diving at Punta de Betín, 11°15′02.1″N 74°13′16.0″W, Santa Marta, Magdalena, Colombia). The isolation was carried out using a modified Zobell medium (1.25 g of yeast extract, 3.75 g of peptone, 18 g of NaCl, 2 g of MgCl_2_, 0.525 g of KCl, 0.075 g of CaCl_2_ and 15 g of agar dissolved in enough distilled water to make 1 l of solution) supplemented with nalidixic acid (50 μg/mL). Genomic DNA extraction was performed using the Quick-DNA Fungal/Bacterial Microprep kit (Zymo Research Corporation, Irvine, CA, USA) following the manufacturer's instructions. The quality of the extracted DNA was verified by agarose gel electrophoresis and quantified using Qubit 1X dsDNA High Sensitivity kit (Invitrogen, Life Technologies, CA, USA).

### 2.2 Whole genome sequencing, assembly and annotation

Whole-genome sequencing was performed by Macrogen Inc. (Korea) using Illumina paired-end sequencing technology. Short read (151 bp) libraries were prepared using TruSeq Nano DNA Library Prep kit (Part # 15041110, Rev. D, Illumina, Inc., San Diego, CA, USA) and sequencing on Illumina NovaSeq 6000 platform. The raw sequence reads were quality filtering, trimming and *de novo* assembled applying the Shovill pipeline v1.1.0 (with default parameters) [2], employing SPAdes as the assembler tool [3]. Contigs shorter than 200 bp were removed. To check the quality of the *de novo* assembly, the genome completeness was analyzed by the BUSCO tool [4] (it reached 99.8%), and the ContEst16S algorithm [5] did not identify contamination in the assembled genome. Genome sequencing and assembly data are available from NCBI BioProject with accession PRJNA798903.

Genome scaffolding was performed using the MeDuSa web server [6] with the reference genomes *Gordonia* sp. SGD-V-85 (RefSeq assembly accession: GCF_001456905) and *Gordonia terrae* (RefSeq assembly accession: GCF_901542405). These genomes were selected considering the results of the OGRI with the *de novo* assembled genome. The *de novo* assembled and scaffolded genomes were compared using the QUAST web server [7]. Genome annotation was done according to the RAST tool kit using the PATRIC service center [8]. To detect and characterize the content of specialized metabolite BGCs, we annotated the genome using the antiSMASH v6.0 tool [9]. A graphical circle map was generated on the CGView server to visualize the annotation results [10].

### 2.3 Phylogeny analysis

The analysis was conducted on both a genome-wide and single gene basis; including 16S ribosomal RNA gene (well established for phylogenetic analysis of bacteria), secA1, and gyrB genes, which have also been used for *Gordonia* phylogenetic analysis with more discriminatory power to identification at the species level [11]. The OGRI were calculated using the TYGS [12] and JSpeciesW [13] web servers. The sequences of 16S rRNA, secA1, and gyrB genes were retrieved from our annotated genome *G. hongkongensis* EUFUS-Z928. Phylogenetic trees for single gene analysis were estimated based on the maximum likelihood method using the IQ-Tree tool [14] (bootstrap values were calculated from 1000 replicates). Phylograms were generated using MEGA v11.0.10 [15]. Whole-genome phylogeny analyses were inferred using the Genome BLAST Distance approach in the TYGS server.

## Ethics Statements

The samples used by this research were of Colombian origin, and they were obtained according to Amendment No. 5 to ARG Master Agreement No. 117 of 26 May 2015, granted by the Ministry of Environment and Sustainable Development, Colombia.

## CRediT authorship contribution statement

**Jeysson Sánchez-Suárez:** Conceptualization, Methodology, Software, Validation, Formal analysis, Investigation, Data curation, Writing – original draft, Visualization. **Luis Díaz:** Project administration, Funding acquisition, Supervision, Writing – review & editing. **Javier Melo-Bolivar:** Software, Validation, Formal analysis, Data curation. **Luisa Villamil:** Project administration, Funding acquisition, Supervision, Writing – review & editing.

## Declaration of Competing Interest

The authors declare that they have no known competing financial interests or personal relationships that could have appeared to influence the work reported in this paper.

## Data Availability

Draft genome of Gordonia hongkongensis EUFUS-Z928 (Original data) (OSF).
